# Can the Mismatch of Measured Pelvic Morphology vs. Lumbar Lordosis Predict Chronic Low Back Pain Patients?

**DOI:** 10.3390/jcm13082178

**Published:** 2024-04-10

**Authors:** Deed E. Harrison, Jason W. Haas, Ibrahim M. Moustafa, Joseph W. Betz, Paul A. Oakley

**Affiliations:** 1 Chiropractic Biophysics NonProfit, Inc., Eagle, ID 83616, USA; 2 Department of Physiotherapy, College of Health Sciences, University of Sharjah, Sharjah 27272, United Arab Emirates; iabuamr@sharjah.ac.ae; 3 Neuromusculoskeletal Rehabilitation Research Group, RIMHS–Research Institute of Medical and Health Sciences, University of Sharjah, Sharjah 27272, United Arab Emirates; 4 Private Practice, Boise, ID 83709, USA; 5 Kinesiology and Health Science, York University, Toronto, ON M3J 1P3, Canada; docoakley.icc@gmail.com

**Keywords:** chronic low back pain, pelvic morphology, spine model, radiography, lumbar lordosis (LL)

## Abstract

**Background**: Measures of lumbar lordosis (LL) and elliptical modeling variables have been shown to discriminate between normal and chronic low back pain (CLBP) patients. Pelvic morphology influences an individual’s sagittal lumbar alignment. Our purpose is to investigate the sensitivity and specificity of lumbar sagittal radiographic alignment and modeling variables to identify if these can discriminate between normal controls and CLBP patients. **Methods**: We conducted a computer analysis of digitized vertebral body corners on lateral lumbar radiographs of normal controls and CLBP patients. Fifty normal controls were attained from a required pre-employment physical examination (29 men; 21 women; mean age of 27.7 ± 8.5 years), with no history of low back pain, a normal spinal examination, no pathologies, anomalies, or instability. Additionally, 50 CLBP patients (29 men; 29.5 ± 8 years of age) were randomly chosen and matched to the characteristics of the controls. The inclusion criteria required no abnormalities on lumbar spine radiographs. The parameters included the following: ARA L1-L5 lordosis, ARA T12-S1 lordosis, Cobb T12-S1, b/a elliptical modelling ratio, sacral base angle (SBA), and S1 posterior tangent to vertical (PTS1). Two measures of pelvic morphology were determined for each person—the angle of pelvic incidence (API) and posterior tangent pelvic incidence angle (PTPIA)—and the relationships between API − ARA T12-S1, API − Cobb T12-S1, and API − ARA L1-5 was determined. Descriptive statistics and correlations among the primary variables were determined. The receiver operating characteristic curves (ROC curves) for primary variables were analyzed. **Results**: The mean values of LL were statistically different between the normal and CLBP groups (*p* < 0.001), indicating a hypo-lordotic lumbar spine for the CLBP group. The mean b/a ratio was lower in the chronic pain group (*p* = 0.0066). The pelvic morphology variables were similar between the groups (*p* > 0.05). API had a stronger correlation to the SBA and Cobb T12-S1 than PTPIA did, while PTPIA had a stronger correlation to the S1 tangent and ARA T12-S1 than API did. While CLBP patients had a stronger correlation of ARA T12-S1 and Cobb T12-S1 relative to the pelvic morphology, they also had a reduced correlation of ARA L1-L5 lordosis relative to their SBA and pelvic morphology measures. API − T12-S1, API − L1-L5, and API − Cobb T12-S1 were statistically different between the groups, *p* < 0.001. Using ROC curve analyses, it was identified that ARA L1-L5 lordosis of 36° and ARA T12-S1 of 68° have a good sensitivity and specificity to discriminate between normal and CLBP patients. ROC curve analyses identified that lordosis ARAT12-S1 < 68° (AUC = 0.83), lordosis ARAL1-L5 < 36° (AUC = 0.78), API − ARA T12-S1 < −18° (AUC = 0.75), API − ARAL1-L5 > 35° (AUC = 0.71), and API − Cobb T12-S1 < −5° (AUC = 0.69) had moderate to good discrimination between groups (AUC = 0.83, 0.78, 0.75, and 0.72). **Conclusions**: Pelvic morphology is similar between normal and CLBP patients. CLBP patients have an abnormal ‘fit’ of their API − ARAT12-S1 and L1-L5 lumbar lordosis relative to their pelvic morphology and sacral tilt shown as a hypolordosis.

## 1. Introduction

Lumbar lordosis (LL) is part of humans’ evolutionary adaptation to upright locomotion; however, a universally agreed upon geometry and magnitude for lordosis remains challenging and problematic. There are several variables that influence the magnitude and geometry of LL across individuals. These confounding variables include the following: (1) advanced age [1,2,3], (2) body mass index [4,5], (3) lumbar vertebra morphology [6], (4) pelvic morphology [7,8,9,10,11,12,13], and (5) sacral morphology [14,15]. Despite the above challenges, several studies have been put forth investigating and developing normative ranges for adult LL values, with many accounting for several of the above five variables [1,4,5,7,8,9,10,11,12,13,16,17,18,19]. Furthermore, several studies have found a correlation between a reduced LL and chronic low back pain (CLBP) [13,16,18,20,21,22,23,24,25]. In contrast, several studies have found no correlation between back pain and the amount of LL [4,5,7,26,27]. However, two recent systematic reviews with meta-analyses have identified that hypolordosis of the lumbar spine is a risk factor for both the presence of back pain [28] as well as the development of back pain [29]. Problematically, in many of the investigations looking at abnormal LL in back pain patients vs. asymptomatic controls, the above five “confounding” variables were not accurately accounted for [28,29]. 

Low back pain has numerous causes, from mechanical, infectious, psychosomatic, inflammatory, and mental-health-related. A differential diagnosis is important for clinicians treating low back pain patients and it is critical to understand the various causes of low back pain and the proper methods to determine the cause. However, mechanically and as elucidated above, a primary contributing biomechanical variable leading to the development and chronicity of CLBP is a hypo-lumbar lordosis [28,29]. One of the strongest determinants of an individual’s LL is thought to be their pelvic morphology [7,8,9,10,11,12,13], where pelvic morphology is defined as the fixed anatomical position of the sacrum inside the ilia in relationship to the bisection point between the center of each femur (hip axis). In 1985, During et al. [7] presented the first pelvic morphology assessment as an anatomical angle of the intersection between a line connecting the mid-sacral endplate of S1 to the hip axis (HA) and a line drawn along the superior endplate of S1. The authors defined this angle as the pelvi-sacral angle and found it to be a primary determinant of the sacral base tilt angle and the magnitude of the distal LL [7]. 

Since During et al. [7], several authors have developed different pelvic morphology measurements, and have investigated the relationship of pelvic morphology variations to both the sagittal plane curves of the spine and sagittal balance (translations of region to region) [8,9,10,11,12,13]. Only three investigations, however, could be located that assessed the pelvic morphology values and LL in normal controls versus CLBP patients [7,13,30]. Problematically, these investigations failed to delineate whether a reduced LL in CLBP patients is due to differences in pelvic morphology between normal controls and CLBP patients. Many authors have previously investigated the differences between pelvic morphology and variations in spine parameters, where these prior studies discuss the normal and compensatory parameters [31,32,33,34,35]. Prior studies of the sagittal spine parameters have confirmed earlier studies on the importance of a normal or average or “neutral zone” that is necessary to reduce the reported suffering from low back pain, spine pathologies, and a reduced quality of life. Sagittal spine balance and the importance of understanding the consequences of a mismatch between pelvic morphology parameters and lumbar spine pathologies is an important endeavor for clinicians and researchers alike [31,32,33,34,35]. 

In 1998, Harrison et al. [20] found that elliptical modeling and LL measures could discriminate between a defined normal group and CLBP patients. However, Harrison et al. [20] did not consider pelvic morphology differences between the groups, nor did they investigate the sensitivity and specificity of their most significant measures to accurately discriminate between the groups. The current investigation revisits the data from Harrison et al. [20] in order to investigate the possibility of pelvic morphology differences between their [20] normal and CLBP groups and to investigate the sensitivity/specificity of discriminatory group variables. Our hypothesis is that pelvic morphology measurements will be significantly different between the normal and CLBP groups such that group lordotic differences can be explained by pelvic morphology variables. Thus, pelvic morphology variables should have a good sensitivity/specificity as a dichotomization variable between the groups.

## 2. Materials and Methods

### 2.1. Patient Data Collection

Previously, differences in elliptical modeling and radiographic measures for the LL in four matched groups of patients (normal controls, acute low back pain patients, CLBP patients, and those with lumbar pathologies) were reported [20]. In the current investigation, we revisit data from two of these groups: the normal controls and CLBP patients. Complete details of these normal controls were previously reported [17]. Our article is a retrospective review of clinical records from two previous publications [17,20] and is exempt from IRB approval under section 45 CFR 46.101(b)(4). See https://www.hhs.gov/ohrp/regulations-and-policy/decision-charts-pre-2018/index.html#c5 (accessed on 4 March 2024). In the normal group, there were 29 men and 21 women with no history of low back pain, a normal physical examination, and no radiographic evidence of pathologies, anomalies, and instability. Their average age was 27 years, average height was 171.5 cm, and average weight was 70.8 kg [17,20].

From the same clinic files as the normal group, 50 CLBP patients (29 males and 21 females) were randomly chosen from a population that matched the group characteristics of the normal controls. Herein, CLBP was defined as greater than 6 weeks of continual pain and/or a recurrent history of pain that caused absence from work or significant modification of daily activities. Inclusion criteria required no abnormalities on lumbar spine radiographs in order to be accurately matched to the normal group. Their average age was 29.5 years, average height was 174 cm, and average weight was 74 kg [20].

### 2.2. Lumbar Modeling

The digitization and elliptical modeling details of these patients have been described previously; however, the relevant details are provided here [19,20]. The lateral lumbar radiographs of all 100 individuals were digitized with a sonic digitizer (GP-9, from Science Accessories Corp., Shelton, CT, USA). In total, 18 points on the vertebral bodies from T12-S1 and the superior aspects of each femur/acetabulum, and pubic symphysis were digitized. The elliptical model extends from the posterior–inferior body corner of T12 to the superior–posterior corner of the body of the first sacral segment and represents the path of the posterior vertebral body co-ordinates between these landmarks [19,20]. Vertebral body x–y co-ordinates were stored in a database, written in FORTRAN 77 to run on a personal computer. The original computer code performed a least-squares approximation of each patient’s LL in the shape of an ellipse. The program iterates to find a best-fit ellipse for each person by passing ellipses, in the least-squares sense, through vertebral x–y co-ordinates along the posterior aspect from T12-S1. For each patient’s best-fit ellipse, the program determined semi-major (a) and semi-minor (b) axes, b/a ratio, and the portion of a quadrant (between 80° and 90°), which the elliptical segment comprised [19,20].

### 2.3. Radiographic Measurements

**Figure 1:**
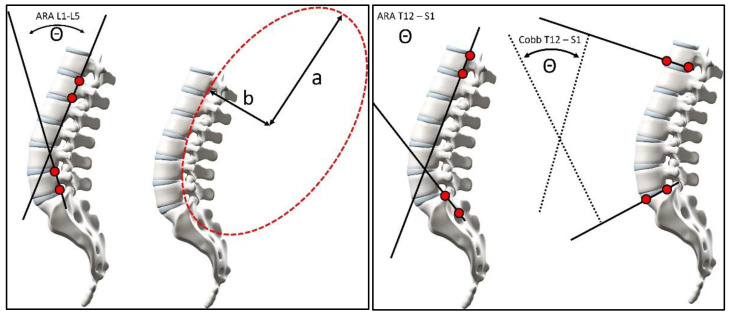
Measurements of lumbar lordosis included in this investigation. (A) Lumbar lordosis measured using the posterior body tangent method ARA L1-L5. (B) Thoraco-lumbar lordosis measurement using the posterior body tangent method ARA T12-S1. (C) Cobb angle of thoraco-lumbar lordosis measurement using the inferior endplate of T12 relative to the sacral surface of S1. (D) The b/a elliptical modelling ratio.

Using the digitized vertebral body x–y co-ordinates, lumbar lordotic alignment variables were calculated. In the previous study, these variables included the following: (1) the intersection of posterior body lines at L1 and L5 forming a global angle of lumbar lordosis (LL) defined as an absolute rotation angle (ARA L1-L5), (2) the intersection of the inferior endplate line of T12 and the superior endplate line of S1 forming a Cobb angle (Cobb T12-S1), and (3) the superior sacral endplate line relative to horizontal or sacral base angle (SBA). See Figure 1 for these measurements.

For the current investigation, several new variables were calculated, including the following: (1) the intersection of posterior body lines at T12 and S1 forming a global angle of thoracolumbar lordosis (ARA T12-S1), (2) the posterior body tangent line of S1 relative to vertical (S1 tangent), (3) angle of pelvic incidence (API), and (4) the posterior tangent pelvic incidence angle (PTPIA). A negative value indicates spinal extension or lordosis. These methods of measurement have been reported to be highly reliable [16,35,36].

### 2.4. Pelvic Morphology

**Figure 2:**
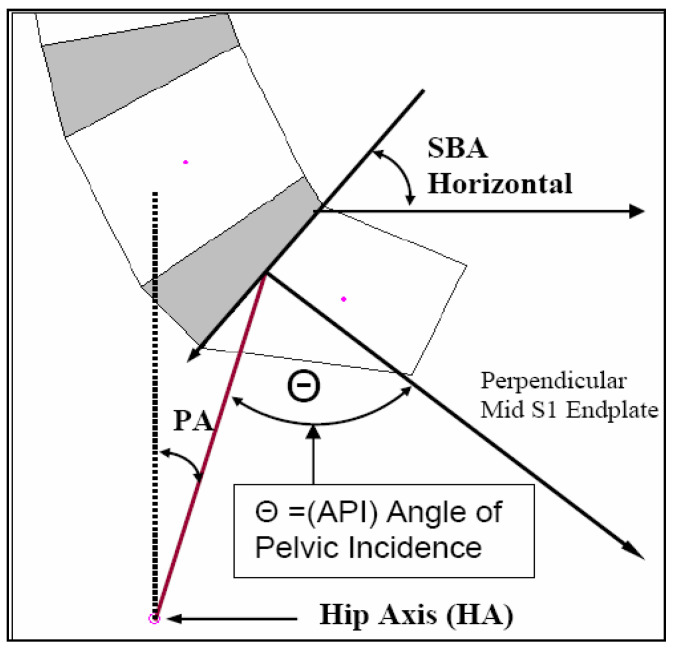
Angle of pelvic incidence (API) [8]. First, a line is drawn across the S1 endplate. Second, a perpendicular line is drawn inferiorly originating at the S1 endplate midpoint. Third, a line connecting the hip axis (bisection of tops of acetabulum in the current study) and the mid-S1 endplate is constructed. The angle Θ between the perpendicular mid-S1 endplate line and the hip axis mid-S1 body line is termed the API. The sacral base to horizontal (SBA) and pelvic tilt (PA) are also shown.

**Figure 3:**
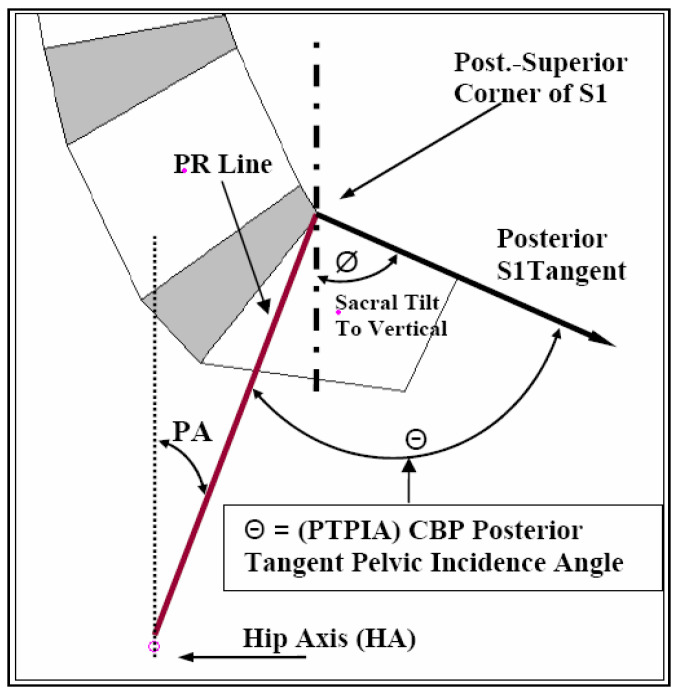
The posterior tangent pelvic incidence angle (PTPIA) is shown as originally developed by Harrison in 2005 [37]. This was adapted from previous methods. First, the PR line is drawn connecting the posterior superior corner of S1 to the hip axis (bisection of the femur heads superior apex points). Next, a line is drawn along the posterior body margin of S1. Then, the angle between the PR line and the S1 posterior tangent line is created as the PTPIA.

For pelvic morphology assessment, two separate measurements were evaluated. In Figure 2, the more recent angle of pelvic incidence (API) from Legaye et al. [8] was calculated instead of the pelvi-sacral angle from During et al. [7]. It is readily observed that the pelvi-sacral angle and the API always add up to 90° and are exactly inversely linearly correlated to each other. The second pelvic morphology angle calculated was the posterior tangent pelvic incidence angle (PTPIA). See Figure 3. In 2005, Harrison et al. [37] originally developed the PTPIA to represent the pelvic morphology relative to the posterior vertebral body tangent method of lumbar lordosis analysis. The API method (Figure 2) utilizes the SBA in construction, while the PTPIA (Figure 3) uses the posterior body of S1. Thus, different correlations and applications might result from these two different pelvic morphology measures. Diebo et al. suggested that the API minus the lumbar lordosis (LL) should be less than 10° (API − LL < 10°) [32] and we wished to analyze this equation relative to our data. Thus, we present an analysis of API − ARA T12-S1, API − ARA L1-L5, and API − Cobb T12-S1 for both our normal and CLBP groups in the results.

### 2.5. Statistical Analysis

The means, standard deviations, maximum and minimum values for LL, sacral tilt, pelvic morphology, and API − ARA T12-S1, API − ARA L1-L5, and API − Cobb T12-S1 alignment variables between the groups were analyzed. Linear correlations were investigated between the continuous variables segregated by pain group (normal and CLBP) and by sex (male or female). The primary correlations sought were between the pelvic morphology variables (API and PTPIA) and measures of LL, sacral tilt, and the elliptical ratio b/a.

To evaluate the sensitivity and specificity of the alignment variables between groups, we analyzed each of the pelvic morphology, lumbar lordotic angles, sacral angles, elliptical b/a ratio, API − ARA T12-S1, API − ARA L1-L5, and API − Cobb T12-S1 with receiver operating characteristic curves (ROC curves). For each ROC curve, we investigated the area under the curve (AUC), the optimum cutoff value between groups, and the sensitivity and specificity at this cutoff. In all cases, the criterion used for choosing the cutoff was minimization represented by the maximum Kolmorgorov–Smirnov (Max K–S) metric and the maximum value of Youden’s index. In the case of multiple cutoff values associated with Max K–S, the largest one is reported. SPSS version 29 was used to analyze the data. 

## 3. Results

### 3.1. Means, Standard Deviation, and Minimum/Maximum Radiographic Parameters

The means, standard deviations, and maximum and minimum values for all variables between the groups are reported in Table 1. There were no statistically significant differences in group means or variances (*p* > 0.05) for both the analysis of variance (ANOVA) F tests and Bartlett’s tests [20]. Since there were no appreciable statistical differences in any of the pairwise relationships between males and females, mean height and weight, and when accounting for the group, the data are presented collectively by groups: normal vs. CLBP. The mean values for all three measures of LL (ARA L1-L5, ARA T12-S1, and Cobb T12-S1) were statistically different between the normal and CLBP groups (*p* < 0.001), indicating a hypo-lordotic lumbar spine for the CLBP group. The mean SBA was not significantly different between the groups (*p* > 0.05) but the posterior tangent of S1 to vertical (PTS1) was (*p* < 0.01). The mean b/a ratio was statistically different between the groups, being smaller in the chronic pain group (*p* = 0.0066). The mean values for the two pelvic morphology variables (API and PTPIA) were not statistically different between the groups. However, all three of the relationships of API − ARA T12-S1, API − ARA L1-L5, and API − Cobb T12-S1 were statistically different between the normal and CLBP groups (*p* < 0.001).

### 3.2. Pelvic Morphology and Matrix of Correlations

API had a stronger correlation to the SBA and Cobb T12-S1 than PTPIA did, while PTPIA had a stronger correlation to PTS1 and ARA T12-S1 than API did. Both API and PTPIA had similar correlations to ARA L1-L5. See Table 2. In terms of the linear correlation by sex, chronic pain male patients had slightly more hypolordosis ARA T12-S1 than chronic pain female patients. However, for both combinations of pain type (normal or chronic) and sex, SBA is positively correlated with API. This correlation was slightly greater for males than for females; despite these slight differences, there were no overt sex differences influencing the main outcomes so the results of the two groups are presented as combined for sex in both normal and chronic pain patients.

### 3.3. Receiver Operating Characteristic Curves

**Figure 4:**
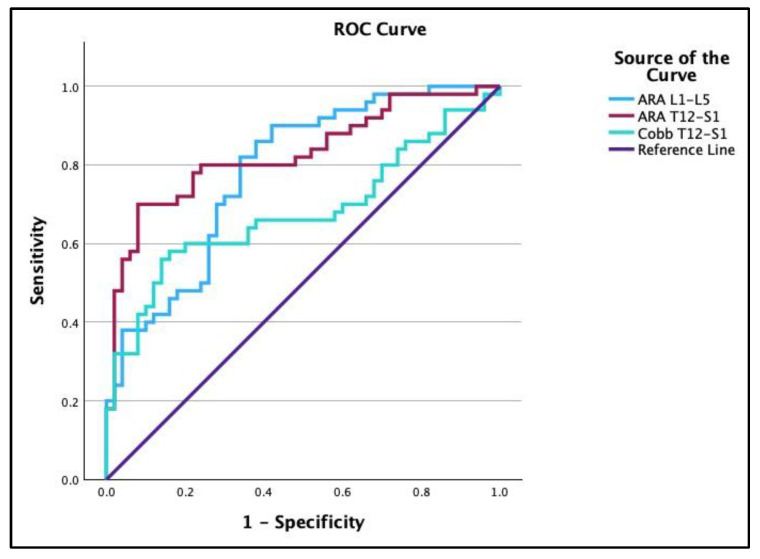
Receiver operating characteristic (ROC) plots. (1) A variable that could distinguish between normal and CLBP groups was ARA L1-L5 (blue): area under the curve (AUC) = 0.78, optimal cutoff value = −36.1°, sensitivity = 0.82, and specificity = 0.66. (2) Variable ARA T12-S1 (red): AUC = 0.83, optimal cutoff value = −67.43°, sensitivity = 0.70, and specificity = 0.92. (3) Variable Cobb T12-S1 (light blue): AUC = 0.68, optimal cutoff value = −56.05°, sensitivity = 0.56, and specificity = 0.86.

**Figure 5:**
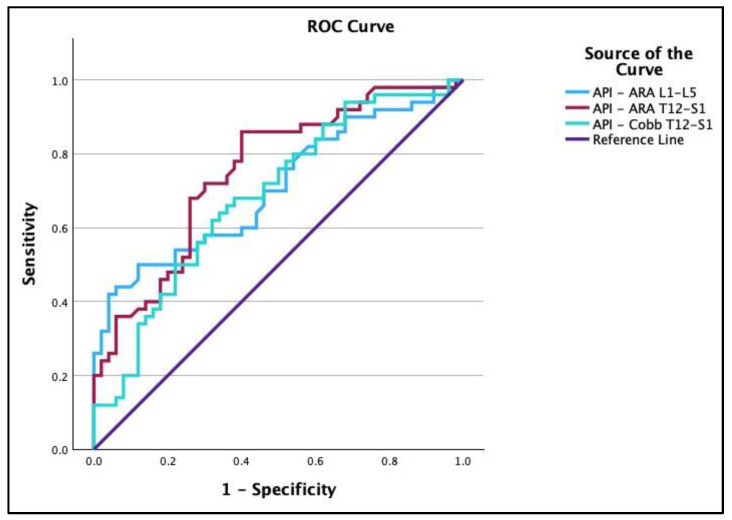
Receiver operating characteristic (ROC) plots for API − ARA T12-S1, API − Cobb T12-S1, and API − ARA L1-L5. These variables showed moderate to good ability to distinguish between normal and CLBP groups. (1) API − ARA T12-S1 (red line): area under the curve (AUC) = 0.75, optimal cutoff value = −17.95°, sensitivity = 0.86, and specificity = 0.6. (2) API − Cobb T12-S1 (light blue line) (AUC) = 0.69, optimal cutoff value = −4.78°, sensitivity = 0.62, and specificity = 0.68. (3) API − ARA L1-L5 (blue line): AUC = 0.71, optimal cutoff value = 35.2°, sensitivity = 0.42, and specificity = 0.96.

To provide a sensitivity and specificity evaluation, receiver operating characteristic curve (ROC) analyses were calculated. In Table 3, three of the variables (ARA T12-S1, Cobb T12-S1, and ARA L1-L5) had a good ability to discriminate between the normal and CLBP groups, while five of the variables (SBA, PTS1, PTPIA, API, and b/a ratio) had no better than random prediction. Figure 4 presents the ROC curves for ARA T12-S1 (67°), Cobb T12-S1 (56°), and ARA L1-L5 (36°) as dichotomous variables between the normal and low back pain groups, where the numbers (67°, 56°, and 36°) are the estimated optimal cutoff values to discriminate between the two groups. ROC plots for API − ARA T12-S1, API − ARA L1-L5, and API − Cobb T12-S1 are shown in Figure 5. API − ARA T12-S1 (red line) showed a good ability to discriminate between normal and CLBP groups: area under the curve (AUC) = 0.75, optimal cutoff value = −17.95°, sensitivity = 0.86, and specificity = 0.6. API − ARA L1-L5 (blue line) showed a reasonable discrimination between the normal and CLBP groups: AUC = 0.71, optimal cutoff value = 35.2°, sensitivity = 0.42, and specificity = 96. API − Cobb T12-S1 (light blue line) showed a reasonable discrimination between the groups: AUC = 0.69, optimal cutoff value = −4.79°, sensitivity = 0.62, and specificity = 68.

### 3.4. Dot Plots for the Relationship of Lumbar Lordosis in Normal vs. CLBP

**Figure 6:**
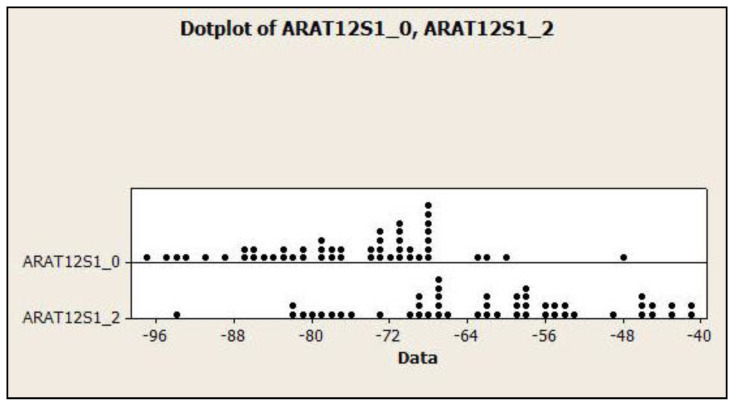

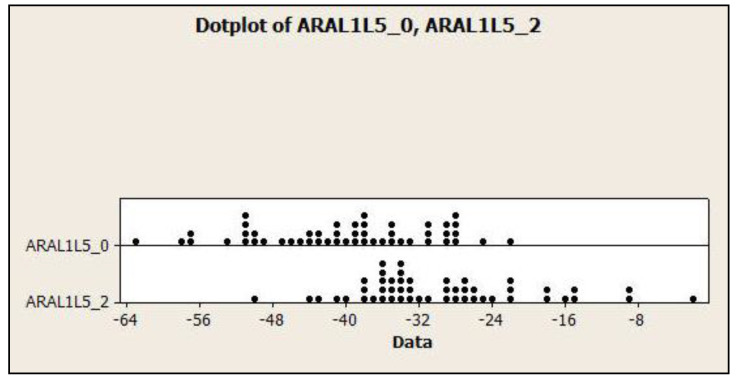
Dot plot addressing the relationship between lumbar lordosis ARA T12-S1 and ARA L1-L5 in the normal and chronic low back pain groups. Group 0 = normal, and 2 = chronic low back pain. Lordosis increases moving to left and decreases to the right. Patients with chronic low back (group 2) tend to have ARA T12-S1 < 68° and ARA L1-L5 < 36°; *p* < 0.001.

In Figure 6, the relationships between LL T12-S1 (ARA T12-S1) and L1-L5 (ARA L1L5) and normal vs. CLBP pain are shown as dot plots. This depiction enables the ROC analysis results to be readily visualized between groups. Patients with CLBP (group 2) tend to have ARA T12-S1 < 68° and ARA L1-L5 < 36°.

## 4. Discussion

In the current investigation, we had hypothesized that differences in sagittal lumbar alignment between matched groups of normal and CLBP patients could be explained by variations in pelvic morphology between the groups. Since the CLBP group had hypolordosis, we expected to find smaller mean values for the API and PTPIA pelvic morphology variables as compared to the normal controls. However, the mean values for the two pelvic morphology variables (API and PTPIA) were not significantly different between normal controls and CLBP patients. Our primary hypothesis must be rejected in favor of the null hypothesis inasmuch as pelvic morphology variables alone do not explain group lordotic differences. Pelvic morphology explains LL in both normal and the CLBP patients depending upon which variables are used to assess the correlation. According to our findings, the chronic low back pain patients exhibit normal pelvic morphology parameters with a larger standard deviation from the mean but have a radiographically measured hypolordosis. Because our normal and CLBP groups have similar pelvic morphology values, but the CLBP patients have hypolordosis of their lumbar spine, our main findings indicate that CLBP patients have an abnormal response (poor fit) of the LL to their pelvic morphology and sacral inclination measures. However, this mismatch of the pelvic morphological parameters compared to lumbar lordosis is only significant for the ARA L1-L5, while the CLBP group actually possesses a statistically significant stronger correlation between the thoraco-lumbar-pelvic-measured lordosis using ARA T12-S1 and Cobb T12-S1. We propose that this is due to the fact that our two groups both have similar values for the SBA, and, previously, it was reported that the CLBP group had a different thoraco-lumbar curvature than the normal group [20]. Still, this finding of a stronger correlation between pelvic morphology and ARA T12-S1 and Cobb T12-S1 in CLBP is surprising and unexpected. However, the weaker correlation between ARA L1-L5 and API, PTPIA, and SBA in the CLBP group would seemingly predict the presence of low back pain in patients with a loss of lumbar lordosis with normal pelvic morphology. As recommended by Diebo and colleagues [32], we presented an analysis of the relative differences between the API and three different lordosis measurements using the following: (1) API − ARA T12-S1, (2) API − ARA L1-L5, and (3) API − Cobb T12-S1; each of these relationships were found to be statistically different between the normal and CLBP groups. The significance of these relationships and findings is detailed below in Section 4.1 and Section 4.2.

Previously, only a few studies have compared pelvic morphology variables in a group of CLBP (non-spondylolisthesis and scoliosis cases) to a control group [7,13,30]. In a study of 52 persons without low back pain compared to 44 patients with one of two lumbar conditions (general low back pain and L5-S1 disc degeneration with LBP), During et al. [7] found clinical differences in the pelvi-sacral angle (pelvic morphology) and distal lumbar curve circular radii in normal controls compared to the CLBP groups. However, their [7] patients were not matched for proper group characteristics. See Table 4. Similarly, Jackson et al. [13] reported the PR-S1 pelvic morphology variable and LL values in 20 controls and 20 matched CLBP patients with degenerative lumbar disc disease. A clinically significant (not statistically) increased PR-S1 value was found in CLBP patients with a concomitant reduced Cobb L1-S1 and T12-S1 LL. Jackson et al. [13] did not comment on the possible group differences in pelvic morphology vs. lordosis in terms of statistical significance; they reported correlations within groups. See Table 4. Lastly, Yoshimoto et al. [30] reported the API pelvic morphology variable and LL values in 150 patients with hip osteoarthritis (HOA) matched to 150 patients with degenerative lumbar disorders. The HOA patients had a statistically significant increased API (58.5 ± 14.0°) compared to the low back disorders group (51.9 ± 13.4°). Although there was no normal control group, the low back disorders group had a significantly reduced LL (L1-S1 Cobb = 35.2 ± 13.2°) and SBA (31.2 ± 10.5°) with the same mean range of the API as found in normal controls reported in the literature (see Table 4). This indicates that the low back group from Yoshimoto et al. [30] had an abnormal reduction or ‘fit’ of the LL to their API as in the current study’s results. 

### 4.1. ROC Curve Analysis

As in the current study’s results, others have found that, on average, CLBP patients have hypolordosis with a reduction in the distal lumbar curve when compared to controls [13,16,18,20,21,22,23,24,25]. The finding of hypolordosis for the lumbar spine in CLBP is consistent with recent systematic reviews as well [28,29]. Typically, however, these studies present their results by comparing the group means and standard deviations [28,29]. This type of statistical analysis does not allow for a complete understanding of the relationship between hypolordosis and pain or if the mean value is an acceptable cutoff value for the determination of normal versus low back pain patients. 

In the current study, we analyzed the ROC curves of the discriminatory pelvic morphology and LL variables between the groups to determine the sensitivity vs. specificity for the entire spread of cutoff values (not just the group means). In Table 3, we found the area under the ROC curve was 0.83 for ARA T12-S1 and 0.78 for ARA L1-L5, whose values are considered to be good classifications between the groups. In Figure 4, the estimated optimum cutoff values for the discrimination of normal vs. CLBP patients for ARA T12-S1 and ARA L1-L5 are 68° and 36°, respectively. These estimated cutoff values of 68° and 36° are more lordotic than the group means of the ones reported in Table 1 for the ARA T12-S1 and L1-L5 in our CLBP group. Diebo et al. [32] recommended the normal relationship between API − LL < 10° as a clinical cutoff value; accordingly, we analyzed the relationship between API − LL for an assessment of the fit of lordosis to pelvic morphology between our two groups. The ROC curves for API − ARA T12-S1, API − ARA L1-L5, and API − Cobb T12-S1 (Figure 5) all show a moderate to good ability to distinguish between normal and CLBP groups, with API − ARA T12-S1 (AUC = 0.75, cutoff value = −18°) being better than API − ARA L1-L5 (AUC = 0.71, cutoff value = 35°), and the least efficient, but still moderate, is API − Cobb T12-S1 (AUC = 0.68, cutoff value = −5°). Our findings are similar in context to those of Diebo et al. [32]; given the differences in measurements, they used the L1-S1 Cobb angle, while the current study used Cobb T12-S1 and posterior body lines on T12-S1, which accounts for the increase in the cutoff value of our finding of −18°. Our use of API − ARA L1-L5 is not comparable to that of Diebo et al. [32] as they included the sacral slope in their angle of LL measurement while L1-L5 does not include this increased angle.

Thus, these values (68° and 36° for ARA T12-S1 and ARA L1-L5) can be recommended as minimum estimated values for LL from T12-S1 and L1-L5 in a CLBP patient who has relatively normal API or PTPIA pelvic morphology values (see Table 1 and Table 4 for normal values). Furthermore, it can be recommended that the difference between API − ARA T12-S1 of −18° is an efficient cutoff value in its ability to discriminate between normal and CLBP groups as well when looking at how the lumbar lordosis fits the pelvic morphology. Significantly, rehabilitation interventions aimed at improving the hypo-lordotic lumbar spine of a CLBP patient have proven to be beneficial in the recent literature [38,39], but further investigations are needed to isolate specific subgroups that respond optimally, and also to determine the ideal treatment frequency and durations for each intervention.

### 4.2. Pelvic Morphology, Sacral Tilt, and Lordosis Mensuration

In 1998, Legaye et al. [8] presented a modification of the pelvi-sacral angle from During et al. [7] which they termed the angle of pelvic incidence (API in Figure 1). The pelvi-sacral angle is the complimentary side of the angle used for the API (API and pelvi-sacral always add up to 90°); as such, these have a perfect inverse correlation and only one is needed. See Figure 1. In the current study, we used a measurement of pelvic morphology (developed by Harrison in 2005) that uses the posterior body of S1 instead of the superior sacral endplate that is often obscured on X-rays for a variety of reasons (degeneration, spondylolisthesis, surgical fusions, etc.) [37]. This measurement was termed the posterior tangent pelvic incidence angle or PTPIA. See Figure 2. The PTPIA is strongly, but not perfectly, correlated to the API. See Table 2.

Our findings show that the PTPIA pelvic morphology variable has a stronger correlation to the S1 tangent angle and ARA T12-S1, while the API pelvic morphology variable has a stronger correlation to the SBA and Cobb angle measurements of LL (Table 2). This finding was expected since the API utilizes the SBA and the PTPIA utilizes the S1 tangent in their construction. Studies in the literature report both types of sacral tilt (SBA [7,8,9,10,11] and S1 tangent [16]) and LL measurements using Cobb angles [8,9,10,11,12,13] and ARA methods [17,19,20]. Therefore, it would seem straightforward that the API pelvic morphology variable should be used when the SBA and Cobb type of angles are measured and the PTPIA pelvic morphology variable should be used when reporting the S1 tangent and ARA’s for the determination of sagittal lumbar alignment. A recent study by Li et al. identified that the cross-sectional area of the lumbar erector spinae muscular, in addition to pelvic morphology, influences the shape and magnitude of lumbar lordosis [40]. Further, the results of Li et al. found that increased proximal lumbar lordosis occurs with increased pelvic morphology (distal lordosis stays the same) which is quite similar to our results of an increased b/a ratio (more circular geometry) with increasing pelvic morphology [40].

Some prior studies have demonstrated the importance of understanding pelvic morphology and the consequences of poor sagittal balance. The correlation to normal spine and sagittal biomechanics has previously been discussed and there have been some discussion regarding the potential pitfalls of having spinopelvic abnormalities that lead to poor sagittal balance. Understanding of the relationship between pelvic morphology and sagittal spine curvatures needs further investigation. Savarese et al. [33] stated in 2020: “Spinal deformity in the sagittal plane has been considered one of the main causes of disability, with a significant impact on health, and reputed by some authors as equivalent to diseases such as cancer, diabetes, and heart disease.” The spinopelvic morphology studies have contributed to measurable average parameters. The importance of our current study expands the understanding of the mismatch between normal pelvic morphology and LL and the consequences for CLBP patients [1,8,9,10,11,12,13,31,32,33,34]. Diebo et al. suggested that the API minus lumbar lordosis (LL) should be less than 10° (API − LL < 10°) [32] and the predisposition of adjacent segment disease after lumbar fusion has been reported with API − LL > 10° [41]. However, the 10° cut point is not so clear-cut. For example, Inami et al. [42] point out that the 10° cut point is only appropriate for APIs in the 50° (±SD) range, whereas, when the API is reduced to 30°, the API − LL equation approaches 0°, while, when the API is large, such as 80°, the API − LL equation approaches 20°. Thus, it becomes clear that it is the correlation between API and LL that matters more than a specific cutoff value in high and low pelvic incidence angles and this concept is similar to the results we report for the ROC curve analysis for ARA T12-S1, Cobb T12-S1, and L1-L5 regardless of the magnitude of a person’s API.

Been et al., in 2019, in an extensive discussion of the importance of a “neutral” zone of pelvic spinopelvic balance, identified it was necessary for proper function and reduction in pathologies. Conversely, they found that “Individuals out of the neutral zone, with accentuated or with decreased pelvic incidence and spinal curvatures, are at a higher risk for developing spinal pathology, back pain, and reduced quality of life” [31]. It is not the intention of the authors to conclude that low back pain is only caused by the mismatch of pelvic morphology and LL. Much of low back pain has a mechanical origin, at least in part [29], and causes include trauma, degeneration, metabolic, psychiatric, inflammatory, infectious, genetic, and postural. Low back pain can also be referred from diseased viscera and vessels. It is important for treating physicians to be aware of these causes of low back pain in order to provide a differential diagnosis. Proper history, orthopedic testing, neurological testing, social history, plain film radiography, and advanced imaging can provide clinical certainty for clinicians and present different avenues of treatment [42,43,44,45]. 

### 4.3. Study Limitations

Because our investigation used a database from a previous publication [20], there were some obvious constraints to the data analysis such as a lack of the standard deviations of certain demographic data such as the height and weight for each of the groups. However, the previous publication did perform proper statistical ANOVAs for age, height, and weight as confounding variables, so that data were used for the interpretation of the current investigation’s analysis. Similarly, detailed information on patient occupation and lifestyle habits was not available; thus, controlling for these variables was not possible. Comparing the results of our pelvic morphology variables in Table 1 to similar studies in the literature [8,9,10,11], it is apparent that our results report slightly larger values for the API. Two obvious reasons exist for this: (1) in approximately 20% of the cases, it was challenging to locate the superior apex of each femur head/acetabulum; and (2) we bisected the superior apex of the femur head/acetabulum instead of the centers of the femur heads for the location of the hip axis (HA). We did this for simplicity; however, doing so caused a slight superior shift of the true HA. Our method of locating the HA, by use of the superior apex of the femur head, has been previously reported [37]. As is the case for many reports in the literature, our cross-sectional study design did not allow us to determine which came first, the low back pain or the abnormal LL. In a unique, prospective study of 403 healthcare workers, Adams et al. [43] found that the loss of LL was a risk factor for the first-time occurrence of low back pain requiring intervention, and this is supported by the more recent meta-analysis on the topic [29]. It is our contention, however, that, regardless of which came first, the abnormal fit of the sagittal lumbar lordotic alignment to the pelvic morphology in CLBP patients is a problem associated with pain and impairment. 

In our current report, we did not have detailed information regarding pain intensity and disability scales for the quantification of CLBP; thus, a correlation of the pain and disability magnitudes with the simultaneous loss of LL was not possible. Other investigators, however, have found that pain, health status scores, and disability are inversely correlated with sagittal lumbar alignment; in other words, the greater the abnormality, the greater the pain and disability [22,24,45]. Additionally, because we used a retrospective sampling of previously reported data, there is some missing examination information in our population such as a detailed analysis reporting the thoraco-lumbar range of motion of each participant [17,20]. However, previously, in a meta-analysis, Sadler and colleagues identified a clear finding of a reduced thoraco-lumbar lateral bending range of motion in chronic lower back pain populations matched to normal controls [29]; thus, we assume that our data would be similar and that the loss of lumbar lordosis is not dictated by any potential altered range of motion that may have existed in our population, but, rather, the amount of the lumbar lordosis dictates an altered range of motion [46,47]. Finally, our study used a relatively young adult population (mean age of 27–28 years) and these data may not apply to pediatric or older adult populations. Future studies are needed to build on the current investigation’s limitations and look at different age groups and types of populations. For example, recent investigations have looked at how altered sagittal postural profiles influence athletic skills and performance in collegiate athlete populations [48], and looking at how the LL fits pelvic morphology in this population might offer further insights to performance, injury potential, and treatment strategies. This is but one possible future avenue for research. 

## 5. Conclusions

The decreased magnitude of pelvic morphology measures do not explain the hypolordosis of the sagittal lumbar spine in CLBP patients compared to a matched group of normal controls. The CLBP patients were found to have an abnormally reduced lumbar lordosis not predicted by reduced pelvic morphology measurements. While CLBP patients had a stronger correlation of thoraco-lumbar-pelvic lordosis (ARA T12-S1 and Cobb T12-S1) relative to pelvic morphology, they also had a reduced correlation of ARA L1-L5 lordosis relative to their SBA and pelvic morphology measures. Using receiver operating characteristic curve analyses, it was identified that the ARA L1-L5 lordosis of 36°, ARA T12-S1 of 68°, API − ARA T12-S1 (−18°), API − Cobb T12-S1 (−5°), and API − ARA L1-L5 (35°) have a good to moderate sensitivity and specificity to discriminate between normal and CLBP patients. Thus, these values for lumbar lordosis are recommended as a minimum value for lordosis when the pelvic morphology values of a patient are within normal ranges as found in control populations. In the future, larger sample sizes are needed to confirm or refute the current findings. Rehabilitation interventions should verify, in patients with normal pelvic morphology and hypolordosis, whether attempting to increase the hypo-lordotic lumbar from spine (ARA L1-L5) to the normal values identified herein in CLBP patients is associated with improved outcomes.

## Figures and Tables

**Table 1:** Means, standard deviations (SD), and maximum and minimum values for all variables used in analysis of normal and chronic low back pain (CLBP) patients.

Variable	Normal Group			CLBP Patients		
	Mean ± SD	Max	Min	Mean ± SD	Max	Min
**Age yrs**	27.7 ± 8.5	52	18	29.5 ± 8	45	15
**Height cm**	171.5 ^$^	^$^	^$^	174 ^$^	^$^	^$^
**Weight kg**	70.8 ^$^	^$^	^$^	74 ^$^	^$^	^$^
**Sex**	29 Males, 21 Females			29 Males, 21 Females		
**ARA L1-L5**	−40.2 ± 9.4°	−22.1°	−62.9°	−29.6 ± 9.6°	−1.6°	−50.3°
**ARA T12-S1**	−76.3 ± 9.9°	−47.8°	−97.3°	−61.9 ± 12.5°	−40.6°	−93.8°
**Cobb T12-S1**	−65.4 ± 9.4°	−44.2°	−83.4 °	−57.0 ± 14.7°	−27°	−85.3°
**SBA**	39.4 ± 7.2°	53.4°	23.9°	38.2 ± 11.6°	61.5°	14.0°
**PT S1**	50.3 ± 7.8°	62.1°	29.7°	43.1 ± 8.8°	74.2°	19.6°
**b/a**	0.389 ± 0.147	0.874	0.150	0.264 ± 0.181	0.747	0.000
**API**	56.8 ± 11°	82.2°	40.6°	58.0 ± 15.6°	95.2°	29.4°
**PTPIA**	73.9 ± 8.9°	92.9°	57.1°	68.4 ± 10.7°	92.4°	45.6°
**API − ARA T12-S1**	−19.5 ± 14.4°	13.8°	−47.6°	−3.3 ± 18°	49.3°	−40.1°
**API − ARA L1-L5**	16.6 ± 11.4°	40°	−7.8°	28.4 ± 16.2°	59.2°	1.5°
**API − Cobb T12-S1**	−8.6 ± 12.9°	18.9°	−33.7°	1.0 ± 14.3°	36.9°	−26.6°

**Note:** Variables are as follows: (1) the intersection of posterior body lines at L1-L5 forming a global angle of lumbar lordosis (ARA L1-L5), (2) the intersection of posterior body lines at T12-S1 forming a global angle of lumbar lordosis (ARA T12-S1), (3) the intersection of the inferior endplate line of T12 and the superior endplate line of S1 forming a Cobb angle (Cobb T12-S1), (4) the superior sacral endplate line relative to horizontal (SBA), (5) the posterior tangent body line of S1 relative to vertical (PTS1), (6) elliptical minor/major axis ratio (b/a), (7) angle of pelvic incidence (API), and (8) posterior tangent pelvic incidence angle (PTPIA). A negative value indicates spinal extension or lordosis. ^$^ Data are missing from original publication records so information could not be retrieved [20].

**Table 2:** The matrix of correlations separated by normal and chronic low back pain groups and respective *p*-values for individual tests of significance that the correlation is zero vs. not zero.

Group	Variable	PTS1	ARA T12S1	Cobb T12S1	ARA L1L5	SBA	API	PTPIA
Normal								
	SBA	0.808 ** p < 0.001	−0.644 ** p < 0.001	−0.764 ** p < 0.001	−0.604 ** p < 0.001			
	API	0.231 *p* > 0.05	−0.047 *p* > 0.05	−0.200 *p* > 0.05	−0.378 ** p = 0.007	0.451 ** p < 0.001		
	PTPIA	0.486 ** p < 0.001	−0.233 *p* > 0.05	−0.136 *p* > 0.05	−0.325 * p = 0.021	0.390 ** p = 0.005	0.858 ** p < 0.001	
	b/a	0.267 *p* > 0.05	−0.369 ** p = 0.008	−0.558 ** p < 0.001	−0.551 ** p < 0.001	0.399 ** p = 0.004	0.269 *p* > 0.05	0.112 *p* > 0.05
**Chronic pain**								
	SBA	0.649 ** p < 0.001	−0.585 ** p < 0.001	−0.897 ** p < 0.001	−0.401 ** p = 0.004			
	API	0.319 * p = 0.024	−0.206 *p* > 0.05	−0.558 ** p < 0.001	−0.242 *p* > 0.05	0.728 ** p < 0.001		
	PTPIA	0.543 ** p < 0.001	−0.348 * p = 0.013	−0.255 *p* > 0.05	−0.268 *p* > 0.05	0.362 * p = 0.01	0.729 ** p < 0.001	
	b/a	0.588 ** p < 0.001	−0.703 ** p < 0.001	−0.612 ** p < 0.001	−0.550 ** p < 0.001	0.464 ** p < 0.001	0.375 ** p = 0.007	0.469 ** p < 0.001

**Note**: Variables are as follows: posterior tangent S1 to vertical (PTS1), sacral base angle to horizontal (SBA), lumbar lordosis T12-S1 (ARA T12-S1 and Cobb T12-S1), lumbar lordosis L1-L5 (ARA L1-L5), angle of pelvic incidence (API), posterior tangent pelvic incidence angle (PTPIA), pelvic radius to sacral base angle (PR-S1), and elliptical minor/major axis ratio (b/a). * *p* < 0.05, ** *p* < 0.01.

**Table 3:** Analyses of area under the curve (AUC), the optimum cutoff, and the sensitivity/specificity at this cutoff for receiver operating characteristic curves.

Groups	Variable	AUC	Cutoff	Sensitivity	Specificity
**Normal vs. Chronic**	ARA L1-L5	0.78	−36.18°	0.82	0.66
	ARA T12-S1	0.83	−67.43°	0.70	0.92
	Cobb T12-S1	0.68	−56.05°	0.56	0.86
	API − ARA T12-S1	0.75	−17.95°	0.86	0.6
	API − ARA L1-L5	0.71	35.2°	0.42	0.96
	API − Cobb T12-S1	0.69	−4.79°	0.62	0.68

**Note:** Lumbar lordosis (ARA T12-S1, ARA L1-L5, and Cobb T12-S1). API − ARA T12-S1, API − ARA L1-L5, and API − Cobb T12-S1 all showed moderate to good ability to discriminate between the normal and CLBP groups. Variables are posterior tangent of S1 to vertical (PTS1), sacral base angle (SBA), angle of pelvic incidence (API), posterior tangent pelvic incidence angle (PTPIA), lumbar lordosis L1-L5 and T12-S1 (ARA L1-L5, ARA T12-S1, and Cobb T12-S1)), and elliptical minor to major axis ratio (b/a). A negative rotational value indicates spinal extension.

**Table 4:** Comparison of current study’s results for pelvic morphology, SBA, and posterior tangent S1 to similar studies in the literature.

Study	P. M. Method	Sample Size and Age in Years	P.M. Normals, Mean ± S.D (°)	P.M. LBP Groups, Mean ± S.D (°)	SBA or S1 Tangent, Mean ± S.D. (°)
During et al. [7]	Pelvi-sacral Angle	+N = 52 C, 44 DD++ and LBP, A = 16–46	41.27 ± 9.98	++DD = 37.4 ± 12.36 ++LBP = 37.3 ± 11.76	SBA C = 40.4 ± 8.8, DD = 37.9 ± 8.2, LBP = 41.3 ± 8.6 Lordosis reduced in DD and LBP subjects
Current Study	1. API 2. PR-S1 3. PTPIA	N= 50 C, N = 50 CLBP Mean A = 27–30	1. 56.8 ± 11° 2. 27.0 ± 9.1° 3. 73.9 ± 8.9°	1. 58.0 ± 15.6° 2. 26.5 ± 13.5° 3. 68.4 ± 10.7°	Both SBA and S1 Tangent. Refer to Table 1 for Results.
Yoshimoto et al. [30]	API	HOA N = 150 A = 61 ± 11, LBP Patients N = 150 A = 58.9 ± 11.7	NA	HOA = 58.5 ± 14, LBP = 51.9 ± 13.4	SBA HOA = 41.4 ± 10.3, LBP = 31.2 ± 10.5 Lumbar lordosis was reduced in LBP
Jackson et al. [13]	PR-S1	N = 20 C A = 27–75, N = 20 LBP A = 26–73	−31.5 ± 8.7 Max = −16 Min = −47	−33.6 ± 11.9 Max = −15 Min = −56	NR T12-S1 Lumbar lordosis was decreased in LBP

Note: +N = number of subjects, A = age range, C = control subjects, ++DD = L5-S1 degenerative disc space narrowing, LBP = nonspecific low back pain. NR = not reported and NA = not applicable. Max = maximum value and Min = minimum value. HOA = hip osteoarthritis patients. P.M. = pelvic morphology.

## Data Availability

Additional pertinent data are available upon request.

## Abbreviations

API	Angle of pelvic incidence
ARA	Absolute rotation angle
CLBP	Chronic low back pain
HA	Hip axis
HOA	Hip osteoarthritis
LL	Lumbar lordosis
PTPIA	Posterior tangent pelvic incidence angle
ROC	Receiver operator characteristic curve
SBA	Sacral base angle
